# Supplementary material for: Optimizing LED Light Intensity and Photoperiod to Promote Growth and Rooting of Medicinal Cannabis in Photoautotrophic Micropropagation
Source: Biology (Basel). 2025 Jun 16;14(6):706. doi: 10.3390/biology14060706 (PMC12189681; doi:10.3390/biology14060706)
Supplement: Supplementary file 1 [file biology-14-00706-s001.zip › biology-3670900-supplementary.pdf]

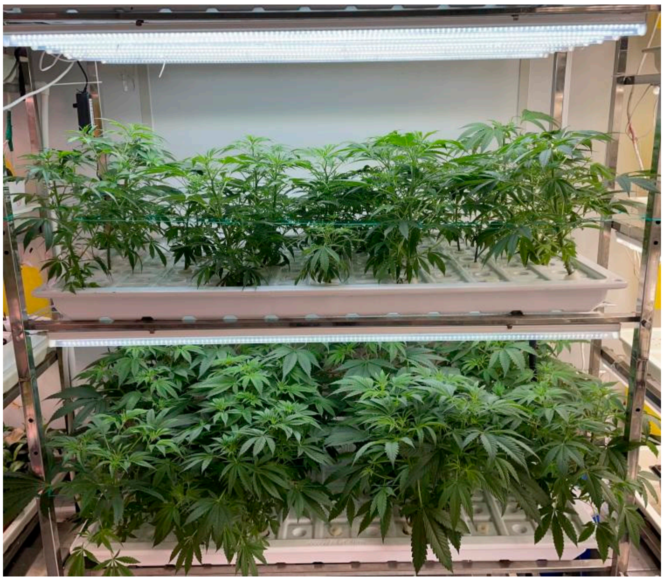

Figure S1. Hydroponic system for mother plants. The system comprises two ABS cultivation beds (length 1200 mm × width 600 mm × height 70 mm) and two 4-mm-thick ABS boards fitted with planting holes (20 mm diameter). The vertical spacing between layers is 70 cm.

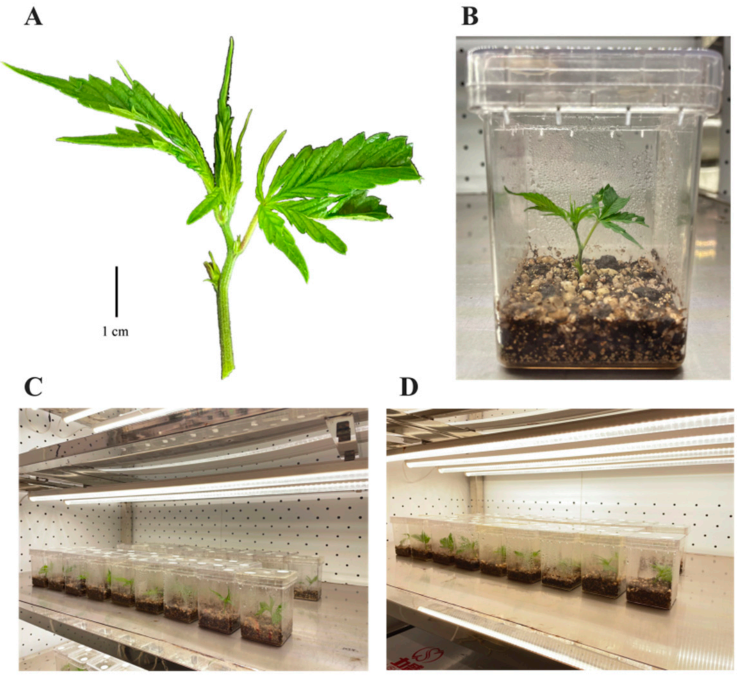

Figure S2. Images of explants and vessel positioning under different light intensities in photoautotrophic micropropagation. A shows an explant before inoculation. B depicts the explant after inoculation in photoautotrophic micropropagation. C and D illustrate the positioning of vessels under varying light intensities within the same photoperiod. Light intensities were controlled by varying the number of LED lamps and moving the position of vessels.

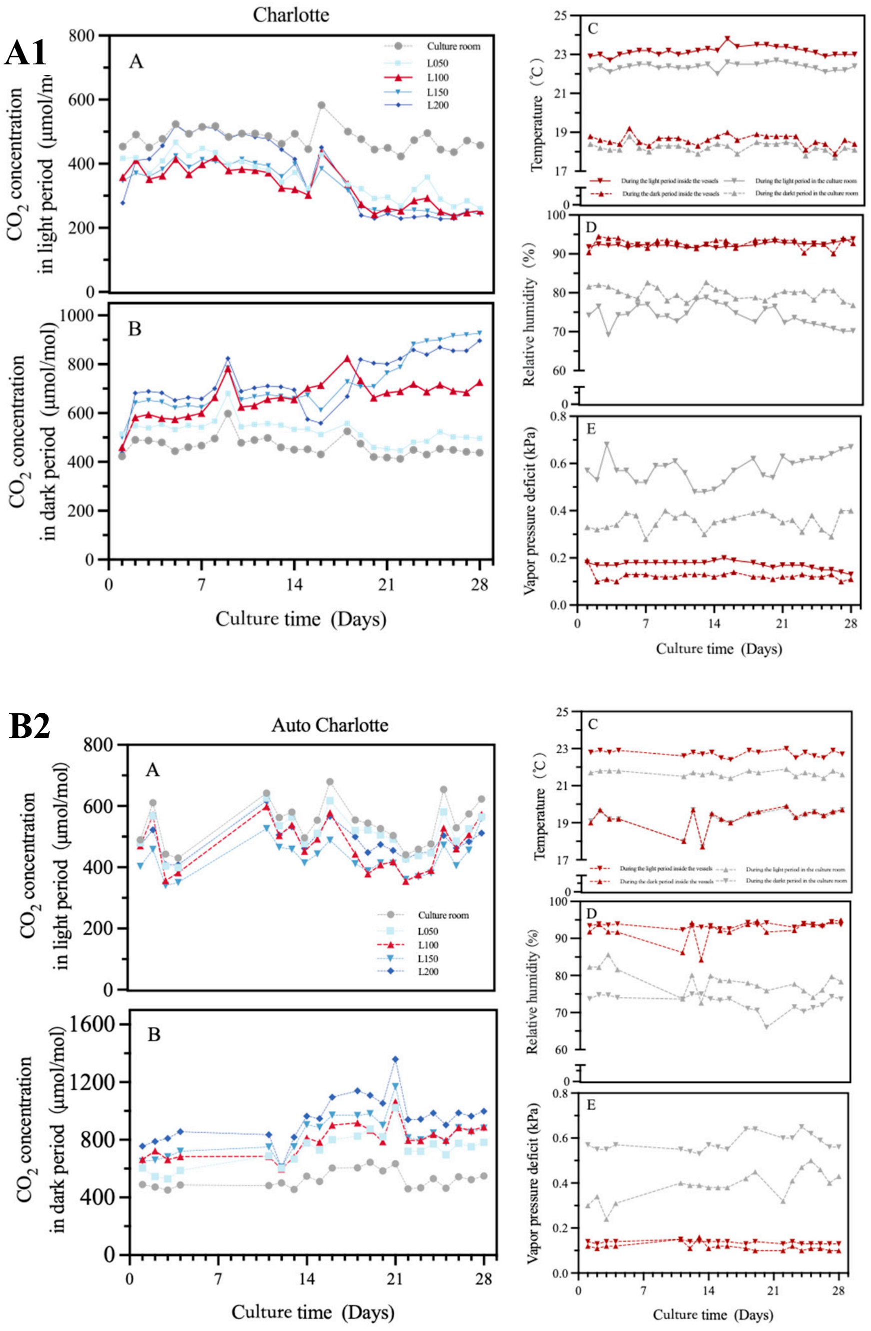

Figure S3. Environmental parameters of the culture room (a controlled environment) and culture vessels for Charlotte (A1) and Auto Charlotte (B1). In the culture room, the temperature was maintained at 22 ± 1°C with 60% ± 5% relative humidity during the light period, and 18 ± 1°C with 70% ± 5% relative humidity during the dark period. CO_2 concentration was not controlled. A1 shows the experimental data for the charlotte cultivar, and B1 shows that for the Auto Charlotte cultivar.

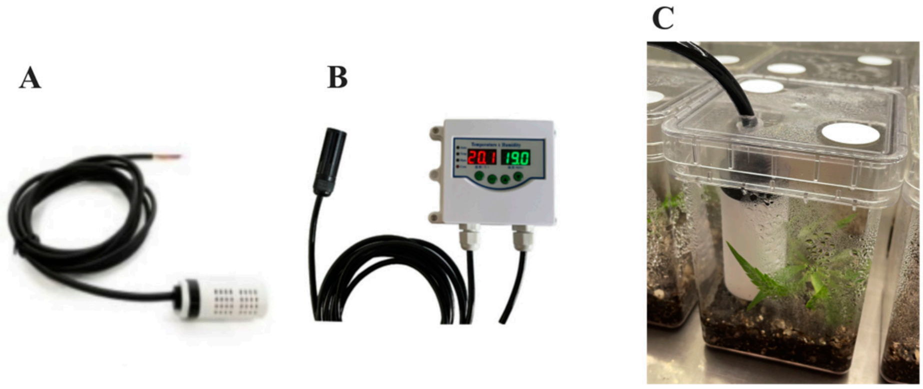

Figure S4. Sensors and installation method in the photoautotrophic micropropagation system. A shows a CO_2 sensor (DCO2-TF series, Dihui Technology Co., Ltd., Beijing, China). B depicts a split-type temperature and humidity sensor (DWS-T5W1-E-S1D, Dihui Technology Co., Ltd., Beijing, China). C illustrates the method of inserting the CO_2 sensor through the GA-7 cap.
